# Integrating ECG Monitoring and Classification via IoT and Deep Neural Networks

**DOI:** 10.3390/bios11060188

**Published:** 2021-06-08

**Authors:** Li-Ren Yeh, Wei-Chin Chen, Hua-Yan Chan, Nan-Han Lu, Chi-Yuan Wang, Wen-Hung Twan, Wei-Chang Du, Yung-Hui Huang, Shih-Yen Hsu, Tai-Been Chen

**Affiliations:** 1 Department of Anesthesiology, E-DA Cancer Hospital, I-Shou University, No. 65, Yida Road, Jiao-su Village, Yan-chao District, Kaohsiung City 82445, Taiwan; ed110880@edah.org.tw; 2 Department of Health and Beauty, Shu-Zen Junior College of Medicine and Management, No. 452, Huanqiu Road, Luzhu District, Kaohsiung City 82144, Taiwan; 3 Department of Anesthesiology, E-DA Hospital, I-Shou University, No. 1, Yida Road, Jiao-su Village, Yan-chao District, Kaohsiung City 82445, Taiwan; hn15537993@gmail.com; 4 Department of Medical Radiology, E-DA Cancer Hospital, I-Shou University, No. 1, Yida Road, Jiao-su Village, Yan-chao District, Kaohsiung City 82445, Taiwan; ed104139@edah.org.tw; 5 Department of Pharmacy, Tajen University, No. 20, Weixin Road, Yanpu Township, Pingtung County 90741, Taiwan; ed103911@edah.org.tw; 6 Department of Radiology, E-DA Hospital, I-Shou University, No. 1, Yida Road, Jiao-su Village, Yan-chao District, Kaohsiung City 82445, Taiwan; 7 Department of Medical Imaging and Radiological Science, I-Shou University, No. 8, Yida Road, Jiao-su Village, Yan-chao District, Kaohsiung City 82445, Taiwan; wang1031@gmail.com (C.-Y.W.); yhhuang@isu.edu.tw (Y.-H.H.); 8 Department of Radiology, Zuoying Branch of Kaohsiung Armed Forces General Hospital, No. 553, Junxiao Rd., Zuoying District, Kaohsiung City 81342, Taiwan; 9 Department of Life Sciences, National Taitung University, No. 369, Sec. 2, University Road, Taitung 95092, Taiwan; twan@nttu.edu.tw; 10 Department of Information Engineering, I-Shou University, No. 1, Sec. 1, Syuecheng Road, Dashu District, Kaohsiung City 84001, Taiwan; wcdu@isu.edu.tw; 11 Institute of Statistics, National Yang Ming Chiao Tung University, No. 1001, University Road, Hsinchu 30010, Taiwan

**Keywords:** ECG, IoT, deep neural network

## Abstract

Anesthesia assessment is most important during surgery. Anesthesiologists use electrocardiogram (ECG) signals to assess the patient’s condition and give appropriate medications. However, it is not easy to interpret the ECG signals. Even physicians with more than 10 years of clinical experience may still misjudge. Therefore, this study uses convolutional neural networks to classify ECG image types to assist in anesthesia assessment. The research uses Internet of Things (IoT) technology to develop ECG signal measurement prototypes. At the same time, it classifies signal types through deep neural networks, divided into QRS widening, sinus rhythm, ST depression, and ST elevation. Three models, ResNet, AlexNet, and SqueezeNet, are developed with 50% of the training set and test set. Finally, the accuracy and kappa statistics of ResNet, AlexNet, and SqueezeNet in ECG waveform classification were (0.97, 0.96), (0.96, 0.95), and (0.75, 0.67), respectively. This research shows that it is feasible to measure ECG in real time through IoT and then distinguish four types through deep neural network models. In the future, more types of ECG images will be added, which can improve the real-time classification practicality of the deep model.

## 1. Introduction

The dangers of surgery and anesthesia have been issues of concern for both physicians and patients for a long time. In addition to the operation itself, there are concerns about the pain of the operation, ignorance of the anesthesiologist, and the method of anesthesia [1]. Hence, it is essential for surgical patients or their families to understand the issues related to surgery and anesthesia.

While we need to engage in a pre-anesthesia assessment, there are many different methods we can choose, including the following: electrocardiogram (ECG), ultrasound (US), a blood test, chest X-ray, and history taking in the clinic [2,3,4]. Except for the methods mentioned above, the last several decades of research have given us helpful information on automated or artificial intelligence methods to assist the pre-anesthesia assessment.

Nowadays, due to the convenience of urban life and the popularity of medical treatment, people can measure their blood pressure in supermarkets and convenience stores close to their homes. However, ECG is more important for real-time measurement of cardiac function than blood pressure. Following the awareness of this, people could easily apply an ECG device anywhere in the future. There must be a specific and popular way to meet the need in terms of such medical concerns. In general, the ECG signal must be interpreted and diagnosed by doctors with their medical expertise, even though people can use the relative machine to obtain their ECG signal. The second reason is that people do not have enough expertise to diagnose themselves at home and doctors cannot make real-time diagnoses at the same time as well. This study aims to investigate whether it is possible to obtain an ECG signal and diagnosis at any time and place. Suppose artificial intelligence (AI) algorithms are used to distinguish pre-discriminate ECG signal patterns measured at home through the Internet of Things (IoT), then people can directly obtain a preliminary report of their ECG anywhere [5,6,7]. For the achievement mentioned above, the main control factor is the signal from the ECG. ECG can be used to understand the activity of the heart and whether the heartbeat is normal and regular [8]. The diseases that doctors can detect through these waveform changes include the following: myocardial infarction, heart displacement, cardiac cystitis, arrhythmia, coronary artery insufficiency, electrolyte metabolism imbalance, atrium or ventricular hypertrophy, etc., [9]. 

In the hospital, a risk assessment before anesthesia is necessary. The ASA Physical Status Classification System has been used for more than 60 years. It allows anesthesiologists and clinicians to evaluate and classify the patient’s physical condition before anesthesia, which can be helpful in predicting operative risk [10]. Later, Dripps (1961) modified the original classification system into a simplified Dripps–ASA model [11,12]. The ASA score is simple and easy to understand. It is a familiar system for anesthesiologists and clinicians to follow. Although this system has been used as a physical status level that guides anesthesiologists through the patient’s overall physical conditions for many years, there are still some risks of having cardiac complications after the surgery.

In cardiac diagnosis, the risk index of cardiac complications is very important. Cardiac complications are risks that require attention when performing non-cardiac surgery. In the literature, simple index derivation and prospective verification can predict the cardiac risk of major non-cardiac surgery. Among stable patients undergoing non-urgent and non-cardiac major surgery, the index can identify patients with a higher risk of complications. The index may help identify candidates for non-invasive techniques or other management strategies for further risk stratification [13]. Lee, et al. studied 4315 patients aged ≥ 50, then developed and validated an index risk for cardiac complications [14]. The outcome of the study shows that the “Receiver operating characteristic curve analysis in the validation cohort indicated that the diagnostic performance of the Revised Cardiac Risk Index was superior to other published risk-prediction indexes”. With the help of the index and non-invasive technologies, anesthesiologists can identify patients with a higher risk of complications after surgery.

ECG is a signal that records the electrical activity of the patient’s heart. It is one of the most common non-invasive technologies used by anesthesiologists with the ECG waveform of ventricular fibrillation (VF) to evaluate the risk before a patient has anesthesia. In the study of “Ventricular fibrillation waveform characteristics of the surface ECG: Impact of the left ventricular diameter and mass” [15] and “Ventricular fibrillation waveform characteristics differ according to the presence of a previous myocardial infarction: A surface ECG study in ICD-patients” [16], it was shown that previous myocardial infarction (MI) could affect the VF waveform and VF characteristics of the surface ECG are not the main consideration of cardioplegia and metabolic status.

## 2. The Study’s Purpose and Methodology

According to the report by the Taiwan Ministry of Health and Welfare [17], in Taiwan, the top three diseases that cause death are malignant tumors, heart disease, and pneumonia. In the past, the role of the physicians could only be to stand next to a patient to observe the vital signs by visual and general medical judgment. However, with the implementation of the prototype concept, doctors will be able to operate active measures they have never used before in the process of curing patients. Doctors could remotely monitor in real time and discuss patients’ vital signs with others from multiple divisions.

**Figure 1:**
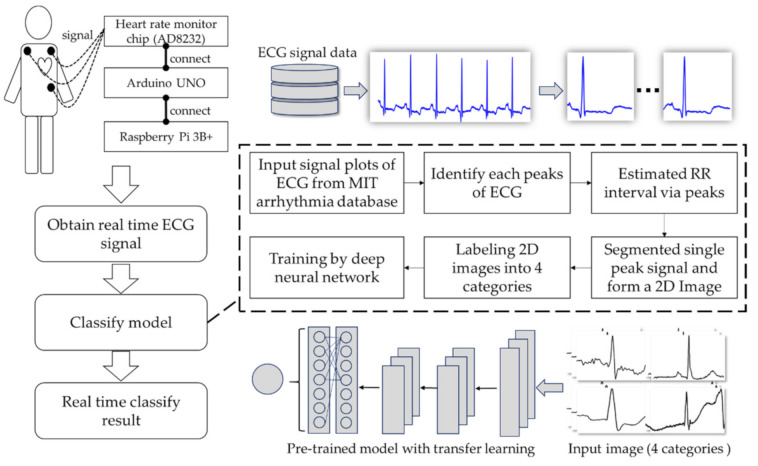
Research structure of the proposed methodology.

The research process first obtains the ECG signal through the developed single-lead prototype and then designs the automatic ECG signal process method. Additionally, it finds the peaks of each signal for a single complete cycle signal cutting; the third step transfers the cut signal as a single JPG image, then uses a deep neural network model to classify the signal category. Therefore, doctors can adopt this device in emergencies as well, e.g., emergency medical technicians (EMTs). Through the remote ECG device, doctors can understand the patient’s status in real time in the process of emergencies. Moreover, during the process of transferring patients to the operating room, anesthesiologists can monitor and check patients’ real-time ECG data on mobile phones at the same time. This research combines an Arduino, a heart rate monitor, and a Raspberry Pi to develop an ECG signal measure prototype that can provide instant ECG waveforms. At the same time, combined with deep learning technology, the waveform type can be automatically identified. The research structure is illustrated below (Figure 1).

### 2.1. A Real-Time IoT ECG Monitor

In order to obtain the real-time ECG signals, the prototype was integrated with IoT hardware devices. The hardware in this study was divided into three major components. One of them is the AD8232 [18], which can be described as a heart rate monitor chip (power voltage: 3.3 V DC; output: analog output). It is suitable for signal conditioning of the cardiac electrical activity from one patient. The second major component is an Arduino Uno, which is an open source development hardware platform. Its open source hardware and software both enable users to develop a wide range of applications. The third one is a Raspberry Pi, which is a Linux-based single-chip computer board. The Raspberry Pi Foundation developed it in the United Kingdom for facilitating basic computer science education with low-priced hardware and free software. The board is characterized by its very light weight, only 42 g, making it ideal for incorporation into portable devices. The tool can be used as a micro-computer that can be plugged into any monitor and accordingly be turned into a fully functioning computer [19]. As mentioned above, its light weight and full function will allow doctors or developers to record data easily. The data could be delivered and accessed by adopting a Wi-Fi schema and cloud storage, then one can obtain analytic results by utilizing a mobile device (iPad, cell phone, etc.). 

The overall operation process is as follows: (1) Following a 10 min rest, ECG electrode pads were applied to three locations of a volunteer (the right arm, left arm, and left leg) in a sitting position. The volunteer did not have any history of heart disease. (2) To acquire about a 9600 baud ECG signal in 600 s, the sampling rate was 360 Hz and the bandwidth was 0.5–40 Hz. (3) The signal flowed from the ECG through the Arduino Uno. In the process, Arduino Uno translated the signal to be analyzed and sent it to the Raspberry 3B+. Furthermore, the combination of the Arduino Uno and Raspberry 3B+ will allow users who are using a remote desktop client to connect to the Raspberry Pi 3B+ and see the data to be analyzed. (4) A total of about 12,000 analog signals were obtained through the process described. Next, the signals were compressed by the fast Fourier transform (FFT) technique. Using FFT removed low-frequency components and an inverse FFT was performed to recover the signal (the noise was defined for a maximum FFT signal × 0.2). (5) The validation of the AD8232 was performed and compared with a clinical ECG by simultaneously measuring 600 s for one individual. We compared the Philips IntelliVue MP70 with the RR interval estimates by the AD8232.

**Figure 2:**
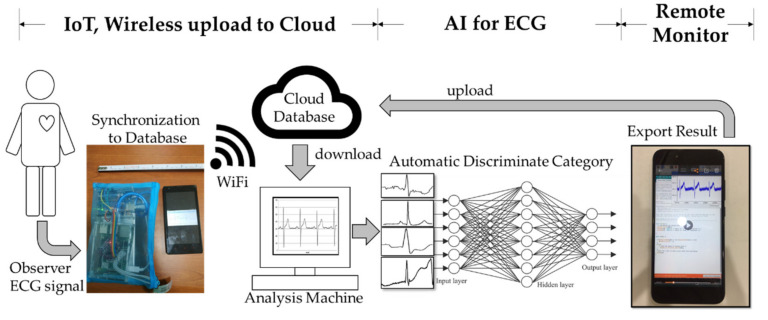
The workflow with combined AI and IoT.

After obtaining the ECG signal through IoT, in order to automate analysis of the ECG signal, AI identification and analysis technology were combined with the present device. First, the ECG signal was synchronized to the cloud database. Then, the analysis machine was used to download the ECG signal, which provided analysis automatically. Next, the data were input into the AI model (through a deep convolutional network) for classification analysis. Finally, the analysis result was output and uploaded to the database storage. Users can view the results in real time on their smart devices. The whole process is shown in Figure 2. The next section will introduce the establishment process of the AI model.

### 2.2. ECG Classified by Deep Learning

**Figure 3:**
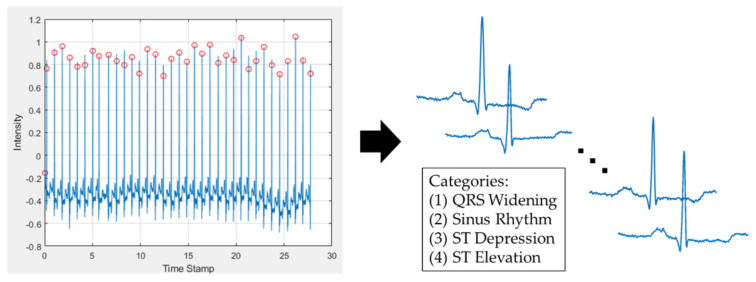
The pre-processing of the ECG signal. On the left is the input signal with automatically found peaks of the R wave. On the right is the segment of each period of the ECG wave.

In order to build a robust classification model, for model training, the ECG data came from publicly available resources in an open database (MIT-BIH Arrhythmia Database) [20,21]. The database contains 48 recordings, each of which has a duration of 30 min and includes two leads. Before training the model, image pre-processing of the ECG signal was performed. First, the peak of the R wave was confirmed, and then the time of the RR interval was calculated. Second, the ECG of the RR interval of one half before and after the interval was cut out (Figure 3). The ECG was composed of a series of wave groups, and each wave group represented every cardiac cycle. The above process was implemented through Waveform Database (WFDB) software. It was suitable for use in the MIT Database. The WFDB could be used for ECG signal processing and analysis, and the software could also be used for viewing, annotation, and interactive analysis of waveform data [21]. A wave group includes a P wave, QRS wave group (QRS complex), and T wave. (1) P wave: The excitement of the heart originates from the sinus node and then moves to the atrium. The P wave is generated by the depolarization of the atria. It was the first wave in each wave group. It reflects the depolarization process of the left and right atria. The first half represents the right atrium, and the second half represents the left atrium. (2) QRS complex: A typical QRS complex consists of three connected waves. The first downward wave is called the Q wave, a high-pointed upright wave after Q wave is called the R wave, and the downward wave after the R wave is called S wave. As they are intricately connected and reflect the ventricular electrical activation process, they are collectively referred to as QRS complexes. This wave group reflects the depolarization process of the left and right ventricles. (3) T wave: The T wave is located after the S-T segment. It is a relatively low and long wave, which is produced by ventricular repolarization.

**Figure 4:**
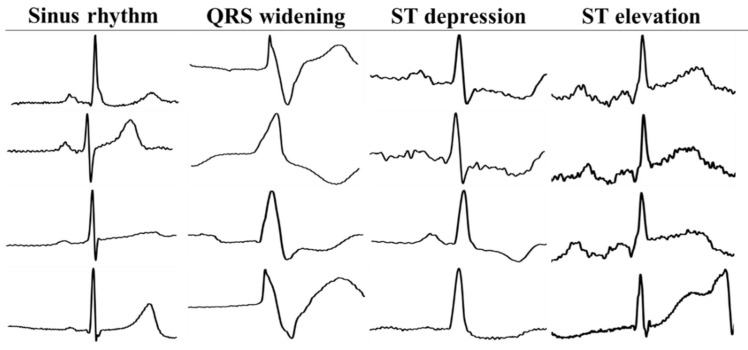
The patterns of QRS widening, sinus rhythm, ST depression, and ST elevation automatically extracted from sequence ECG signals.

According to the MIT-BIH Arrhythmia Database, the four patterns of the signal of an ECG involved in this study include the sinus rhythm (*n* = 19,751), QRS widening (*n* = 21,377), ST depression (*n* = 7163), and ST elevation (*n* = 5899) (Figure 4). The four patterns of the ECG were made into 2D images, and these 2D images were used for building the convolution neural network (CNN) models. The classifier was split with fifty percent each as training and testing datasets. For example, the ST elevation signal had 5899 images. The training versus testing sets had 2950 and 2949 images, respectively. The classification model is trained by the training set and tested by the testing set. These models were created by pre-trained CNNs, which were ResNet [22], AlexNet [23], and SqueezeNet [24]. The numbers of 2D ECG images of the four patterns are shown in Table 1.

### 2.3. The Deep Neural Network (DNN)

For human beings, image-based information is the most intuitive. Therefore, a need for image interpretation has quickly risen in the scientific community. A computer with intelligence capable of interpreting image information is required. Image information has opened many doors and has led us to the field of deep learning. Deep learning is an artificial intelligence function that imitates the workings of the human brain in processing data and creating patterns for use in decision making [25,26]. Deep learning is a subset of machine learning in artificial intelligence (AI) that has networks capable of learning unsupervised from data that is unstructured or unlabeled. Additionally, it is known as deep neural learning or deep neural networks. Convolution neural networks (CNNs) are a subset of deep neural networks, and have attracted a lot of attention in recent years and are used in image recognition [27,28,29]. They are often used to extract features and identify the surrounding environment to build a deep network. As the convolutional neural network structure has more convolution and pooling layers than traditional neural networks, instead of simply extracting data for calculation, it can handle translation, rotation, and distortion. Furthermore, it also retains shape and spatial information to enable image processing. The advantage of this is that it is more convenient and faster than traditional neural networks. In addition, it can reduce the risk of overfitting and neural training parameters [30]. Its feature processing ability has an important application value in the fields of image classification and computer recognition. 

The architecture of a common CNN includes an input layer, a convolution layer, a rectification linear unit layer (ReLU layer), a pooling layer, a fully connected layer, a softmax layer, and a classification layer. In this study, transfer learning technology was utilized to modify the pre-trained CNN models. Transfer learning refers to the use of pre-trained models to modify and train new samples [31,32]. In the training process of convolutional neural networks, a large number of samples are needed to avoid the problem of overfitting; however, in the medical field, few large datasets can be used, so there is a bottleneck in training. The training model can solve the problem of limited datasets. The parameters of the pre-trained model were trained through a large database. The model only needs to fine-tune the parameters for new samples and does not need to retrain the entire model using random initialization parameters.

This study utilized pre-trained ResNet, AlexNet, and SqueezeNet models. All of them are popular for image classification in CNNs. In ResNet, the input image dimension was 256 × 256. It has fifty-three convolution layers, one max-pooling layer, and one fully connected layer, followed by a softmax output layer. In AlexNet, the input image dimension was 256 × 256. It has five convolution layers, three max-pooling layers, seven ReLU layers and two fully connected layers, followed by a softmax output layer. In SqueezeNet, the input image dimension was 256 × 256. It has twenty-six convolution layers, three max-pooling layers, and twenty-six ReLU layers.

## 3. Results

The presented prototype machine was used and compared with routine ECG in a hospital. It simultaneously measured 600 s and yielded 722 RR intervals for one individual. The ECG signal between our prototype and routine ECG was verified by a Wilcoxon signed-rank test. The results were not significantly different (*p* = 0.058, the threshold for significance was 0.05). In addition, an MA plot was adopted to investigate the difference in RR intervals. It showed the RR intervals between our prototype and routine ECG within ±0.1 s.

In the deep neural network, the MIT-BIH database was used to establish the classification model. It could achieve this study’s main aim, which was real-time remote ECG monitoring and classification of ECG patterns by DNN models. The experimental result is shown below (Table 2 shows the predicted confusion table). The designed framework of using ResNet, AlexNet, and SqueezeNet included 50% of the dataset for the training model and 50% of the dataset for the testing model. The ST elevation set had the smallest number of images (i.e., each set was used, with 5899 images). The training and testing sets had 4 × 2950 and 4 × 2949 images, respectively. The DNN classification result is shown in Table 3 and Table 4. Table 3 presents the metrics of each class between the three frameworks. Additionally, Table 4 presents each framework’s detailed result. 

**Figure 5:**
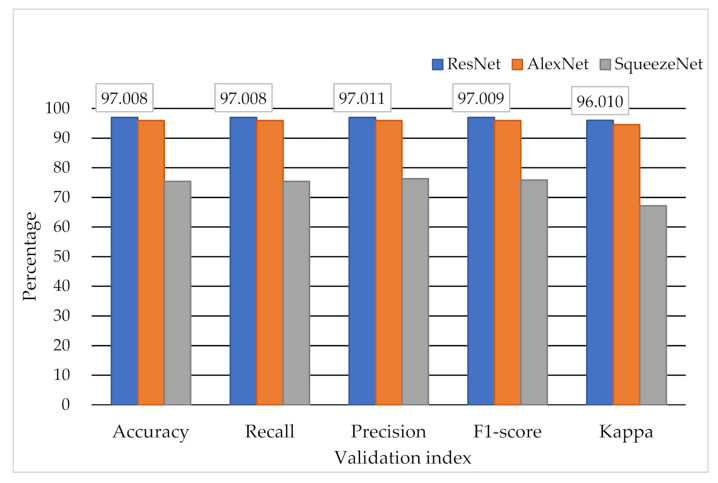
The classification results by different DNN models.

The results provided by the testing set (*n* = 2949) are shown in Table 4. The best accuracy, recall, precision, F1-score, and kappa statistics were 0.97, 0.97, 0.97, 0.97, and 0.96 with, respectively, by using the ResNet model. The testing results among ResNet, AlexNet, and SqueezeNet showed high accuracy and agreement when using ResNet for classifying the 2D signal of the ECG. The histogram of validation values between ResNet, AlexNet, and SqueezeNet is shown in Figure 5. In this study, the accuracy was defined as the correct rate of the overall judgment of the model. The precision was defined as the proportion of actual positives in the case of positive predictions. The recall was defined as how many positives are correctly judged under the condition of actual positives. The F1-score was defined as a comprehensive evaluation index used when both precision and recall are important (F1-score = (2 × precision × recall)/(precision + recall)). The kappa statistic was defined as the consistency of the classification results and actual results.

To compare between the evaluation metrics of the proposed model and recent studies on automated detection and classification [33], the results are presented in Table 5. The proposed method can focus on rhythm, QRS widening, ST depression, and ST elevation categories with an accuracy of 0.97. 

## 4. Discussion

This study indicated that with the employment of an Arduino Uno, Raspberry Pi, AD8232 heart rate monitor, and deep neural network, it is possible to carry out the real-time remote monitoring of variation in patients’ ECGs. Currently, cell phones are an indispensable tool for everyone. A lot of things used to only be done by computers, and now can be accomplished via smartphones. Therefore, if doctors can take advantage of mobile devices to monitor patients’ vital signs, there is no doubt that it will benefit both patients and doctors.

This created prototype was low cost, operated in real time, and reliable. It is available for both experimental research and ECG data collection. Lowering the cost can speed up and promote research. The device realizes the concept of IoT. Health care specialists can control all kinds of instruments via cell phones. Additionally, the real-time vital signs from the instruments can be sent back to specialists concurrently. Hence, this allows doctors to administer the corresponding treatment in time. The presented device can be applied to a patient with a resting heart rate of 60–100 bpm, but it may not be accurate with arrhythmia or tachycardia.

Such a prototype may not replace the current laboratory or hospital equipment, but the IoT concept we proposed is aimed at enabling doctors to discuss and analyze the same patient’s condition in real time. Moreover, the application can be promoted in medical colleges or senior high schools, so students can experience and learn the ECG principles. Meanwhile, ECG is always an important signal for humans. ECG is a transthoracic technique that records the electrophysiological activity of the heart in units of time. In addition, ECG captures and records it through electrodes on the skin. This is a non-invasive way of recording and could diagnose heart rhythms.

With the increase in long-term ECG records, the demand for ECG analysis has relatively increased. This prototype can extract the ECG signals in the time domain and frequency domain of important information. The post-processing algorithm provides extracted data that are compatible with a clinical ECG monitor. Such a prototype cannot replace the current hospital equipment, but the IoT concept we proposed is aimed at enabling doctors or researchers to discuss and analyze ECG data at a lower cost.

## 5. Conclusions

ECG is the best method to measure and diagnose abnormal heart rhythms. There are two conditions used to diagnose abnormal heart rhythms. First, when the electrocardiographic conduction tissue is damaged. Second, when the heart rhythm changes due to electrolyte imbalance. In the diagnosis of myocardial infarction, it can specifically identify the area of myocardial infarction. In this study, the obtained classification results show that the proposed DNN model can be used to classify four categories of disease from ECG images. Meanwhile, the classification results of normal and abnormal ECGs can be used as one of the indicators of risk assessment for anesthesia.

The study created a prototype machine to enable real-time IoT monitoring. It will allow physicians to obtain data to identify cardiac disease from ECG images. The presented device can be applied to a patient with a resting heart rate of 60–100 bpm. This is a protocol and conceptual device for monitoring possible arrhythmia. Consequently, additional different samples will generate a more accurate conclusion and general concepts. Meanwhile, arrhythmia in both different heart rates and different morphologies will be studied to test the presented device in future work. In conclusion, this prototype machine may allow real-time remote monitoring and automatic recognition of the different rhythm classes. It may assist physicians in long-term monitoring or urgent situations. This may be a subject for further study.

In the future, a complete system is to be built up according to the achievements mentioned above. Doctors can monitor the ECG in real time through IoT and mobile devices with the complete system. This immediate and effective information can provide medical staff with the most effective information to facilitate subsequent relevant diagnosis and treatment. Moreover, it can also apply to the fields of home health care (HHC) and long-term care (LTC) to provide medical information generated by IoT devices at home. LTC supports a range of services for patients with chronic illnesses. Physicians from multiple divisions can monitor the important information in real time and provide appropriate advice to the patients’ family. 

## Figures and Tables

**Table 1:** The numbers of 2D images of four patterns of ECG.

Label	Number of 2D ECG Images
QRS Widening	21,377
Sinus Rhythm	19,751
ST Depression	7163
ST Elevation	5899

**Table 2:** The confusion table with true positives, true negatives, false positives, and false negatives in four categories predicted from ResNet, AlexNet, and SqueezwNet.

Label (True Condition)	Predicted by ResNet/AlexNet/SqueezeNet			
	QRS Widening	Sinus Rhythm	ST Depression	ST Elevation
QRS Widening	2803/2754/2187	30/56/128	47/95/276	69/44/358
Sinus Rhythm	39/64/626	2904/2855/2122	3/26/86	3/4/115
ST Depression	54/43/311	61/44/183	2827/2857/2034	7/5/421
ST Elevation	29/76/197	6/6/75	5/21/126	2909/2846/2551

**Table 3:** The metrics for each class and three different models.

Categories	Classifying Model: ResNet/AlexNet/SqueezeNet		
	Recall	Precision	F1-Score
QRS Widening	0.950/0.934/0.742	0.958/0.938/0.659	0.954/0.936/0.698
Sinus Rhythm	0.985/0.968/0.720	0.968/0.964/0.846	0.976/0.966/0.778
ST Depression	0.959/0.969/0.690	0.981/0.953/0.807	0.970/0.961/0.744
ST Elevation	0.986/0.965/0.865	0.974/0.982/0.740	0.980/0.973/0.798

**Table 4:** The framework of Resnet, AlexNet, and SqueezeNet with transfer learning results.

Index	ResNet	AlexNet	SqueezeNet
Image Dimensions	256 × 256	256 × 256	256 × 256
Deep Layers	177	25	68
Accuracy	0.97008	0.95897	0.75398
Recall	0.97008	0.95897	0.75398
Precision	0.97011	0.95907	0.76291
F1-Score	0.97009	0.95902	0.75842
Kappa	0.96010	0.94529	0.67198

**Table 5:** Comparison of proposed scheme with existing methods.

Author	Category	Sample Size	Method	Accuracy
B. Pourbabaee et al. [34]	2 classes	200	CNN + K-nearest neighbor	85.33
A. Hannun et al. [35]	12 classes	91,232	DNN	97.00
M. Javadi et al. [36]	3 classes	15,566	Negative correlation learning method	96.02
Y. Li et al. [37]	1 classes	129	CNN	94.30
Q. Zhang et al. [38]	2 classes	220	CNN	93.50
P. Georgios et al. [39]	5 classes	8528	CNN + BiLSTM	95.90
Presented Method	4 classes	5899	DNN	97.00

## Data Availability

Not applicable.
